# Crystallisation Kinetics and Associated Electrical Conductivity Dynamics of Poly(Ethylene Vinyl Acetate) Nanocomposites in the Melt State

**DOI:** 10.3390/nano12203602

**Published:** 2022-10-14

**Authors:** Gertrud Stalmann, Aleksandar Matic, Per Jacobsson, Davide Tranchida, Antonis Gitsas, Thomas Gkourmpis

**Affiliations:** 1 Department of Applied Physics, Chalmers University of Technology, 412 96 Gothenburg, Sweden; 2 Department of Physics, University of Gothemburg, 405 30 Göteborg, Sweden; 3 Department of Physics, Philipps-Universität Marburg, 35037 Marburg, Germany; 4 Innovation & Technology, Borealis Polyolefine GmbH, 4021 Linz, Austria; 5 Innovation & Technology, Borealis AB, 444 86 Stenungsund, Sweden

**Keywords:** percolative networks, crystallisation dynamics, dielectric hysterisis

## Abstract

Nanocomposite systems comprised of a poly(ethylene vinyl acetate) (EVA) matrix and carbon black (CB) or graphene nanoplatelets (GNPs) were used to investigate conductivity and crystallisation dynamics using a commercially relevant melt-state mixing process. Crystallisation kinetics and morphology, as investigated by DSC and SEM, turn out to depend on the interplay of (i) the interphase interactions between matrix and filler, and (ii) the degree of filler agglomeration. For the GNP-based systems, an almost constant conductivity value was observed for all compositions upon cooling, something not observed for the CB-based compositions. These conductivity changes reflect structural and morphological changes that can be associated with positive and negative thermal expansion coefficients. GNP-based systems were observed to exhibit a percolation threshold of approximately 2.2 vol%, lower than the 4.4 vol% observed for the CB-based systems.

## 1. Introduction

Nanocomposites consisting of polymers and high aspect ratio nanofillers such as graphene, graphene nanoplatelets (GNP) and carbon nanotubes (CNT) have been attracting attention recently due to their outstanding overall performance [1,2,3,4,5,6]. Traditionally, systems consisting of insulating polymers such as polyethylene with conductive carbon black (CB) nanoparticles as a filler are used for semiconductive nanocomposites. This type of system has been successfully developed and is well-established in the industry. However, a high loading of CB is necessary in order to obtain high enough conductivities for many applications (~10^3^ Ω·cm), which results in more brittle material and processing difficulties. It is therefore desirable to find fillers that achieve the required conductivity at a lower loading, so that the materials mechanical properties are closer to those of the neat polymer. GNPs consist of two-dimensional graphite layers, and modern preparation methods can ensure thicknesses that range from one to several atoms [1,2,3]. Consequently, they possess very interesting electrical [7,8,9,10,11,12], mechanical [12,13,14] and rheological [15,16,17] properties. The incorporation of such carbonaceous fillers in a polymer matrix leads to some degree of electrical conductivity, with the resulting system exhibiting a percolation-type of behaviour [18,19,20,21,22]. For traditional nanocomposites containing carbon black particles, it has been reported that the observed electrical conductivity experiences a hysteresis effect upon thermal cycling [23] while undergoing phase transitions. Traditionally, the nature of this hysteresis effect has been explained via a positive temperature coefficient (PTC) leading to a drastic decrease in conductivity close to the melting temperature of the composition, followed by a conductivity increase upon further heating in the melt (negative temperature coefficient; NTC) [24,25]. However, the lack of PTC and NTC upon cooling of some composite systems is not fully understood. It is thus important to investigate which dynamics in the nanocomposite cause this hysteresis effect observed in the conducting behaviour. Furthermore, an understanding of the underlying processes is helpful for designing enduring semiconductive materials for long-term applications.

The presence of nanofillers in the polymer matrix can also affect the overall morphology and dynamics of the system [26,27]. Non-isothermal crystallisation studies for a number of different polymer/filler combinations, including one-dimensional fibres, two-dimensional platelets and three-dimensional particles, have been reported [28,29]. Nanofillers that are of sizes similar to the polymer crystals have been reported to influence crystallisation [26], and in some cases, act as nucleating agents [29]. Here it must be said that the manner in which the filler is incorporated in the matrix has been seen to play a vital role to the overall dispersion and subsequent properties [5,30,31,32,33]. Methods that allow for the exfoliation of the filler and limit its aggregation once incorporated in the matrix have been seen to offer significant advantages. Unfortunately, most of these methods involve various solvation and sonication stages which render them commercially unattractive and plagued with potential health and safety dangers for large-scale production. The ideal case, in terms of financial viability, production simplicity and risk mitigation, is for systems where the filler is incorporated in the melt with minimal use of pre-production steps. These melt mixing methods have been seen to lead to systems with significant aggregation and limited dispersion of the filler (especially in the case of high aspect ratio), but a number of preparation methods focusing on the process [34,35,36,37] and the filler treatment [38,39] have been suggested that aim to tackle these shortcomings.

Poly(ethylene vinyl acetate) (EVA) is one of the most commonly used elastomeric polyethylene copolymers; it has been studied extensively with respect to its electrical, mechanical and thermal properties. It can be produced in both tubular and autoclave reactors and has a structure dominated by long-chain branches in a manner similar to low-density polyethylene (LDPE) [40].

In this paper, we report on the electrical and thermal behaviour of nanocomposites based on carbon black and graphene nanoplatelets with poly(ethylene vinyl acetate) (EVA). First, the effects of filler content and type on the electrical conductivity response were investigated by temperature-dependent dielectric spectroscopy. The local polymer dynamics were analysed further to elucidate the degree of interaction between filler and matrix, as in the vicinity of the particles, chain packing would be initiated, leading to mobility changes. As the absence of specific interactions was established, the morphology and its associated crystallisation and melting were subsequently probed by scanning electron microscopy (SEM) and differential scanning calorimetry (DSC) at different cooling rates. The difference in the electrical percolation threshold of the two composites was traced in the lamellar arrangement, as reflected by different lamellar thicknesses and a decrease (CB systems) or increase (GNP systems) in crystallisation temperature. We propose that the temperature dependence of conductivity depends not only on positive or negative temperature coefficient mechanisms, but also on the favourable or not overall energy reduction, depending on the special localisation and size of aggregates. Finally, the effect of VA content on conductivity was also studied.

## 2. Materials and Methods

The polymers used in this study were EVAs with varying vinyl acetate (VA) content were obtained from ExxonMobil, Irving, TX, USA. For clarity, the different polymers are indicated in this study as EVA followed by a number corresponding to the VA percentage of the material. For example, EVA9 is a material with 9% VA. A summary of their molecular properties is presented in Table 1.

GNPs were obtained from XG Sciences, USA. The average thickness was 6 nm and diameter 25 μm. The carbon black used in this study was a standard commercial N550-type with a mean particle size of 56 nm, Brunauer–Emmett–Teller (BET) specific surface area value of 42 m^2^/g and OAN value of 121 cc/100 gm.

All values from technical data sheets were provided by the suppliers.

All composites, in the range 0.1–22 vol% with GNP and 1.7–30 vol% with CB, were prepared by melt mixing using a Plast-Coder PLE331 Brabender GmbH (Duisburg, Germany) kneader. The system was preheated to 423 K before EVA was added. During filling, the rotation speed was kept to 10 rpm, with the polymer melt homogenised for 2 min at 20 rpm. The speed was further decreased to 10 rpm while the conductive filler was added, with the final composite mixed for 8 min at 20 rpm.

Broadband dielectric spectroscopy (BDS) was performed using a Novocontrol Alpha Analyzer in a frequency range of 10^−2^ to 10^7^ Hz, at different temperatures in the range 243.15–393.15 K, with an error of ±0.1 K, at atmospheric pressure and under a nitrogen atmosphere. For selected temperatures, frequency scans were also performed to investigate the local and ion dynamics. The sample cell consisted of two silver-coated electrodes 40 mm in diameter and the sample had a thickness of about 1 mm. The complex dielectric permittivity ε* = *ε*′ − i*ε*″, where *ε*′ is the real and *ε*″ is the imaginary part, is generally a function of frequency, *ω*, temperature, *T*, and pressure, *P* [41], although here only the frequency and temperature dependencies were investigated. The complex dielectric conductivity σ* can also be calculated from the complex dielectric function ε* as σ* = iωε_f_ε*, (*ε_f_* is the permittivity of free space, 8.854 pF/m), where conductivity can also be analysed in a real and an imaginary part: σ* = *σ*′ + i*σ*″. This means the conductivity data are effectively an alternative representation of the permittivity, albeit focusing on different features of the dielectric behaviour, as becomes apparent later on. The analysis was carried out using the empirical equation of Havriliak and Negami [42]

(1) $$\frac{\varepsilon *(\omega ,T,P)-\varepsilon_{\infty }(T,P)}{\Delta \varepsilon (T,P)}=\frac{1}{{\left[1+(i\omega \tau_{HN}(T,P))a\right]}^{\gamma }}$$

where *τ_HN_*(*T*,*P*) is the characteristic relaxation time in this Equation, Δε(*T*,*P*) is the relaxation strength of the process under investigation, *ε*_∞_ is the dielectric permittivity at the limit of high frequencies and *α*, *γ* (0 < *α*, *αγ* ≤ 1) describe, respectively, the symmetrical and asymmetrical broadening of the distribution of relaxation times. The relaxation times at maximum loss (*τ*_max_) presented herein were analytically obtained by fitting the relaxation spectra with the Havriliak–Negami (HN) Equation as follows:

(2) $$\tau_{\max}=\tau_{HN}{\left[\frac{\sin\left(\frac{\pi \alpha }{2(1+\gamma )}\right)}{\sin\left(\frac{\pi \alpha \gamma }{2(1+\gamma )}\right)}\right]}^{-1/\alpha }$$

An alternative study of conductivity over temperature, referred to herein as the “thermal cycling”, was performed. First, the sample was annealed at 393 K for 10 min to erase the thermal history. Then, 2.5 thermal cycles of alternating heating and cooling between 293 K and 393 K were done at a constant temperature gradient of 5 K/min. A single frequency of 100 Hz was chosen, so that the temperature during each measurement could be assumed constant.

Differential scanning calorimetry (DSC) was performed with a TA Instrument Q1000 under a nitrogen atmosphere. Heating and non-isothermal measurements were conducted in the temperature range 213–393 K at rates of 5, 7, 10, 20 and 30 K/min, respectively. The relative degree of crystallinity as a function of time can be determined by integrating over the heat flow with appropriately chosen boundary conditions.

(3) $$X_{T}=\frac{\int_{T_{0}}^{T}\left(dH_{c}/dT\right)dT}{\int_{T_{0}}^{T_{\infty }}\left(dH_{c}/dT\right)dT}$$

(4) $$t=\left(T_{0}-T\right)/\beta$$

where *X_T_* is the relative degree of crystallinity at the temperature *T*, *H* is the heat flow, *β* is the temperature gradient and *t* is the time. The integration boundaries are chosen as the onset and the end of the crystallisation. From the relative degree of crystallinity, the half time of crystallisation can be found, which is related to the rate of crystallisation.

Scanning electron microscopy (SEM) images were collected with a FEI Quanta 200F on samples that were etched for 15 min using a solution of 1 wt% KMnO_4_ in 86% H_2_SO_4_.

## 3. Results and Discussion

### 3.1. Electrical Behaviour

**Figure 1:**
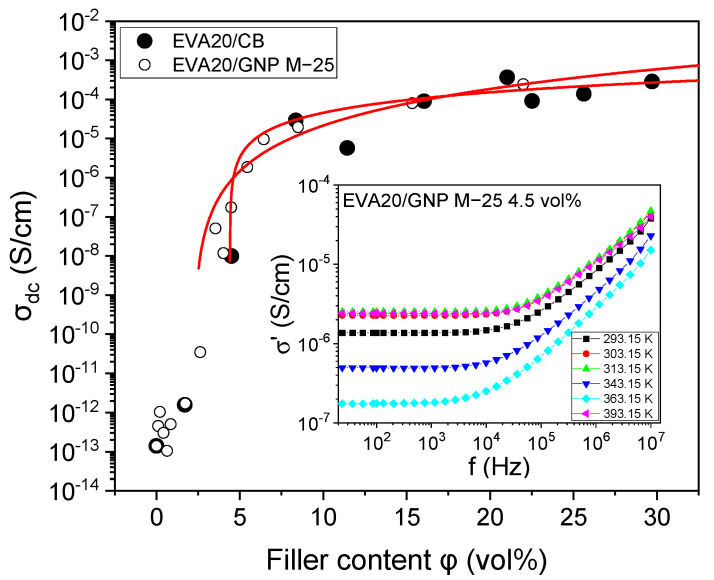
Conductivity at 23.7 Hz and 363.15 K as a function of filler loading for EVA20/CB and EVA20/GNP M−25, indicating the percolative behaviour. The solid lines are fit to Equation (5). In the inset, an example is presented of frequency-dependent conductivity as a function of temperature for the EVA20/GNP M−25 filled to 4.5 vol%. The frequency-independent DC conductivity can be extracted from the plateau observed at low frequencies.

For the determination of the high-temperature percolation threshold, the temperature of 363.15 K was chosen, where the systems over the entire composition range exhibit DC conductivity (*σ_dc_*) at the limit of low frequency (23.7 Hz). In Figure 1, the conductivity as a function of filler loading for the different compositions based on EVA20 can be seen along with the frequency-dependent conductivity for the EVA20/GNP M−25 system filled to a level of 4.5 vol%.

The conductivities follow a percolation-type behaviour described by the power law Equation

(5) $$\sigma =\sigma_{0}{\left(\varphi -\varphi_{c}\right)}^{t}$$

where *σ_0_* is a pre-exponential factor that is dependent on the conductivity of the filler, the network topology and the types of contact resistance. The terms *φ* and *φ_c_* correspond to the filler concentration and the critical concentration at the transition (also known as percolation threshold), while *t* is the critical exponent. A percolative behaviour between 5 vol% and 20 vol% can be observed for the CB-based compositions, with the main increase of several orders of magnitude at a loading of *φ_c_* = 4.4 ± 0.2 vol% (*t* = 1.2 ± 0.8; *σ_0_* = 6 × 10^−6^ S/cm). It must be noted, however, that a very high conductivity of the order of 10^−4^ S/cm is not reached until a loading of approximately 20 vol%. Similar percolation threshold values for untreated CB particles for EVA28 (28% vinyl acetate) have been reported [43]. For a high-density polyethylene (HDPE) system containing similar CB to the one used in this study, a percolation threshold of approximately 18 vol% is reported [44]. This value is slightly lower than the one reported in this study, and the differences can probably be attributed to the use of different processing methods, as well as the use of a different polymer. HDPE has significantly fewer short and long-chain branches than EVA, thus allowing the polymer to pack around the filler in a different way. This packing may or may not affect the polymer dynamics and overall conductivity, as we see below.

The percolation threshold observed for the GNP fillers is lower than the one observed for CB using the same preparation procedures, namely, *φ_c_* = 2.2 ± 0.2 vol% (*t* = 2.6 ± 0.1; *σ_0_* = 1 × 10^−7^ S/cm). The equivalent DC conductivity of the 20 vol% sample is of the order of 10^−4^ S/cm, in good agreement with previously reported data [45]. Nevertheless, the value reported in this study is significantly higher than values reported in the literature [44,46,47,48] using a wide range of mixing techniques. The main reason for this discrepancy can be attributed to the poor mixing and extensive aggregation and agglomeration of the platelets that lead to a significant reduction of the effective aspect ratio of the overall filler. For a similar system of poly(ethylene butyl acrylate) (EBA) with GNP prepared using similar techniques, the percolation threshold has been reported to be 6.9 vol% [35]. These two polymers (EBA and EVA20) have similarities with respect to their detailed chain conformation, both having a polyethylene-based structure dominated by extensive long-chain branching. Still, despite these similarities, the percolation thresholds reported are significantly different, something that can be attributed to differences in the distribution of the polar groups along the backbone and the resulting polarity differences. In the case of EVA, the VA polar groups are distributed mainly homogeneously along the backbone, whereas in the case of EBA, the BA polar groups exhibit an uneven distribution due to the differences in the reaction rate in comparison to ethene during polymerisation [49,50]. 

**Figure 2:**
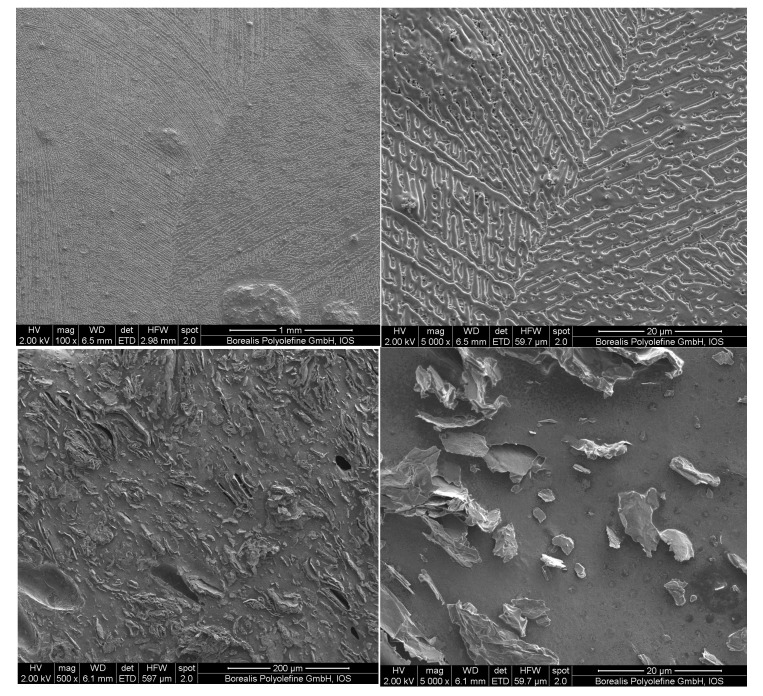
SEM micrographs of EVA20/CB (**top**) and EVA20/GNP M−25 (**bottom**).

In Figure 2, we can see scanning electron microscope (SEM) pictures of EVA20 with CB and GNP. From these pictures, it is clear that the level of agglomeration and aggregation observed in the EVA20/GNP system is fairly large, leading to an inefficient dispersion, something that reflects the relatively high electrical percolation threshold observed. From these pictures, the CB-based compositions appear to adopt a more spherulitic morphology, whereas the GNP-based systems appear to adopt a more disordered two-dimensional arrangement.

Nominal values for bulk conductivity of CB and GNP, as provided by the suppliers, are 10^−1^–10^−2^ S/cm and 10^2^–10^7^ S/cm, respectively. Despite the large conductivity difference between the two fillers, the CB-based compositions exhibit higher overall conductivity than the equivalent GNP-based ones for loadings far above the threshold (Figure 1). The main reason behind this behaviour can be attributed to the poor dispersion achieved via simple melt mixing, something reported extensively in the literature [1,5]. Despite the low percolation threshold achieved, poor dispersion of the GNP particles/aggregates destroys the overall potency of the high aspect ratio filler for high conductivity.

**Figure 3:**
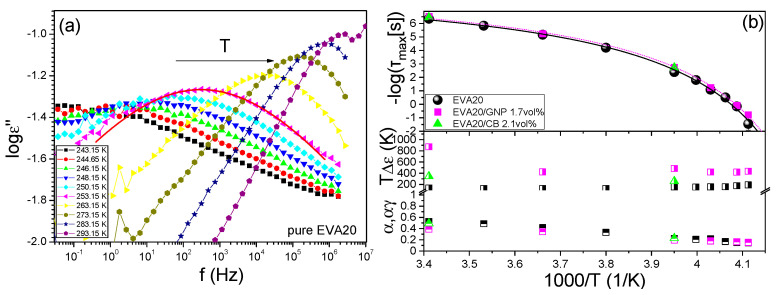
(**a**) Dielectric loss of EVA20 as a function of frequency at atmospheric pressure obtained on cooling. The solid line is an example of the fit to the HN function (2). (**b**) Top: Arrhenius relaxation plot of the segmental (α-) process of EVA20 (black circles) and characteristic composites with GNP (violet squares) and CB (green triangles) close to the conductivity threshold. The data have been fitted with the VFT equation. Bottom: The temperature dependence of the HN shape parameters α = αγ (squares) and TΔ*ε* (circles) of the three samples.

We now turn to the details of the dielectric behaviour of the composites. EVA can be classified as a type-B or C polymer in Stockmayer’s classification [51], as the permanent dipoles are attached perpendicular to the main chain and have some degree of freedom, permitting internal rotation. This dipole can be measured with accuracy employing dielectric spectroscopy and be used as a sensitive probe of segmental relaxation. Figure 3a gives dielectric loss spectra for the segmental relaxation of EVA measured under isothermic conditions. It is apparent that a superposition of the isobaric relaxation spectra is not possible, in contrast to the isothermal spectra at different pressures, as reported in the literature [52], i.e., the system is thermorheologically complex at the segmental level. This is most probably due to the fact that the thermal energy is not great enough to break the predominant intermolecular hydrogen bonding. The intensity of the relaxation spectra of the four EVAs with different VA content follow the trend shown in previous studies [53]. However, the interphase between the polymer matrix and the conductive filler is rather sharp, as implied by the fact that the relaxation times of EVA are not affected by the presence of the fillers. In the small loading domain, up to 1.7 vol% GNP and 2.1 vol% CB, the spectra are identical to those of the pure EVA; thereafter, the high conductivity above the conductive threshold masks the weaker dipolar relaxation. This has the implication that the conductive pathway solely consists of units of the conductive filler, leaving the polymer matrix unaffected and thus retaining the desirable thermomechanical properties, allowing a good polymer design.

In Figure 3b, the relaxation times for the segmental (α-) process were extracted and plotted in the Arrhenius representation as a function of the reciprocal absolute temperature. The relaxation times display the usual strong non-Arrhenius T dependence that conforms to the Vogel–Fulcher–Tammann (VFT) Equation [54,55,56]:

(6) $$\tau =\tau_{0}\exp\left(\frac{DT_{0}}{T-T_{0}}\right)$$

where *τ*_0_ is the relaxation time at the high temperature limit (−log*τ*_0_ = 8.7 ± 0.2), *T*_0_ (=227 ± 1 K) is the “ideal” glass temperature and *D* (=1.6 ± 0.2) is a dimensionless parameter which provides the apparent activation energy as *DT*_0_. The calorimetric glass temperature corresponds to a relaxation time of ∼100 s and was found equal to *T*_g_ = 242 ± 2 K and 241 ± 2 K for EVA20 and EVA/GNP 1.7 vol%, respectively. Those temperatures are in very good agreement with the *T*_g_ obtained from DSC (Supporting Information, Figure S1). In the same figure, the shape parameters of the Havreliak–Negami equation are provided as a function of temperature. It can be seen that the process becomes broader and gains strength with decreasing temperature, reflecting the increasing steric hindrance effect on the movement of the vinyl acrylate side dipole. The analysis of the relaxation spectra of the EVA20/GNP 1.7 vol% (Figure S2) is depicted along with that of the pure EVA20 and EVA20/CB 2.1 vol% in Figure 2b. Whereas the relaxation times and shape parameters remain practically unchanged, the intensity of the process has increased in the composites. This implies the absence of specific interactions between the matrix and the filler. The intensity increase on the other hand is in agreement with the observations of Hallouet et al., which could be attributed to phenomena such as increased ionic mobility close to the matrix–filler interphase [57].

### 3.2. Thermal Cycling

**Figure 4:**
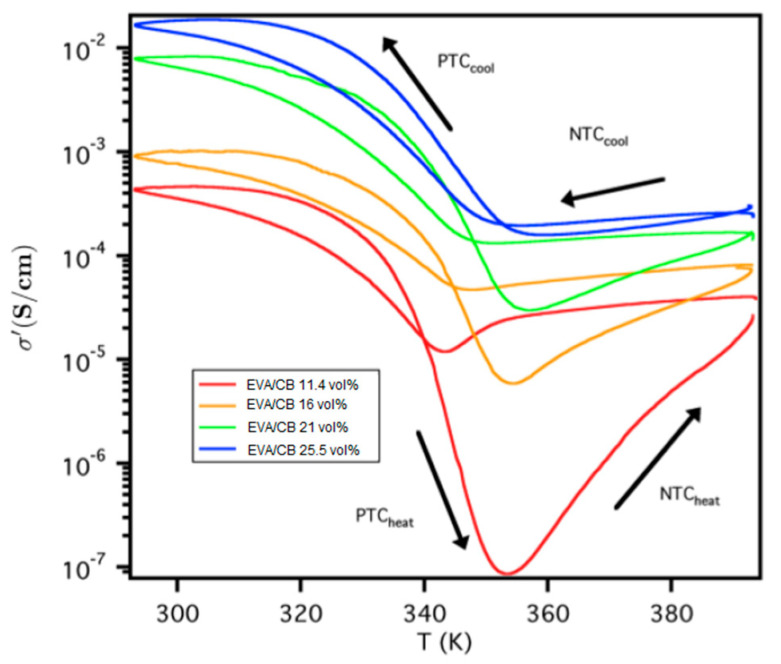
Thermal cycling effect on conductivity for EVA20/CB systems of different loadings. The arrows indicate the cooling and heating curves via the positive and negative temperature coefficients.

In Figure 4, the temperature dependence of the conductivity for various EVA20/CB composites can be seen. The system’s conductivity does not reach upon heating the same level as before the melting process, exhibiting a limited degree of “self-healing”. Traditionally, the temperature dependence of the conductivity is treated via positive (PTC) and negative (NTC) temperature coefficients, respectively [58]. PTC is evoked where the conductivity decreases with increasing temperature and NTC where conductivity increases upon heating. In Table 2, the PTC and NTC values observed for the EVA20/CB system can be seen.

PTC_heat_ and NCT_cool_ can be associated with a measure of the conductive pathway destruction during melting and crystallisation. A large coefficient corresponds to a significant pathway disruption, whereas a small coefficient value corresponds to minimal pathway disruption, as manifested by the interparticle distances that reduce the probability of hopping conduction. NTC_heat_ and PTC_cool_ describe particle rearrangements after the phase transition. These rearrangements can originate by particle diffusion on the polymer matrix or the reduction of the available volume during crystallisation, since the conductive CB particles and agglomerates are located primarily in the amorphous region of the matrix [59]. At high filler loadings, the system is saturated and, although volume changes during phase transitions occur, the average particle position, as seen by the integrity of the conductive pathway, remains almost constant. The change of PTC_cool_ as a function of filler loading, on the other hand, is less distinct. It can be associated with the reduction in crystallinity that occurs as the filler amount increases. At low loading, the higher crystallinity causes the available volume for the CB particles to be reduced due to their preferred location in the amorphous part of the polymer. Due to this, the effective density of the CB particles is increased so that the network formation is improved, thus increasing the conductivity. In other words, for the CB composites, the kinetics are probably more important than the absolute conductivity.

There are very few reports on temperature coefficients for CB-based composites. For a system of cross-linked ethylene butyl acrylate/CB, the conductivity changes are reported to originate from changes in the interparticle distances (gaps), which on their part are attributed to the existence of a polymer restricting the particle movements [58]. This gap opening and closing mechanism, however, does not explain the differences observed during cooling and heating in thermal cycling. Similarly, for an uncross-linked EVA28/CB system, PTC and NTC are reported to occur below and above 353 K, respectively [44], values in good agreement with the ones observed in this study.

**Figure 5:**
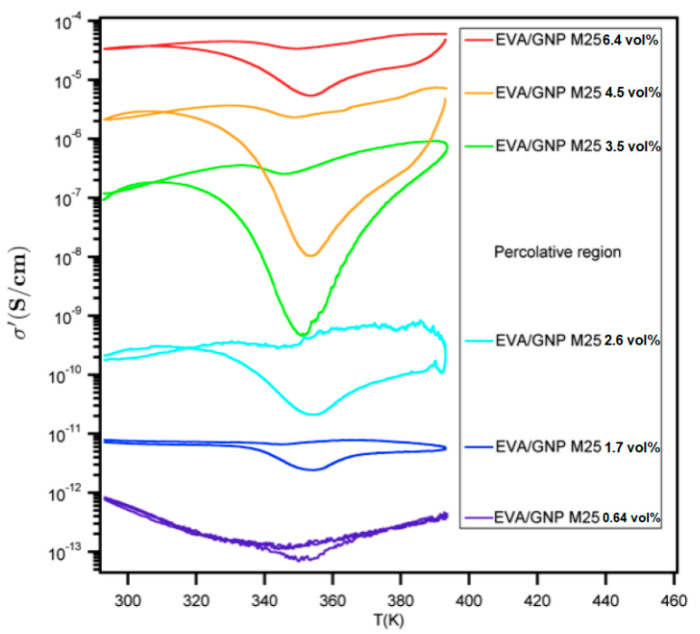
Thermal cycling effect on conductivity for the EVA20/GNP compositions of different filling. For all systems both below and above the indicated percolative region, there appears to be a limited change in conductivity upon cooling.

In Figure 5, the effect of filler loading for the EVA20/GNP composites on conductivity as a function of temperature can be seen. In a way similar to the CB-based composites, the system undergoes a series of phase transitions as it passes the melting and crystallisation temperatures during thermal cycling. The GNP-based compositions, though, appear to have a lack of significant conductivity change upon cooling across the entire loading range. Even at loading of 0.4 vol%, a nearly flat conductivity trend upon cooling is visible, indicating the importance of the polymer–filler interactions. From Figure 5, several important properties can be seen: the sharp increase in conductivity in the region of the threshold and the lack of conductivity changes upon cooling can be observed at loadings significantly below the percolative region. In order to evaluate the nature of this effect, the PTC and NTC values were used in a manner similar to that used for the CB-based composites (Table 3).

Both PTC and NTC are larger upon heating than upon cooling. For loadings of 2.6–4.5 vol%, there appears to be a maximum between heating and cooling, suggesting a maximal hysteresis effect; this is something seen from the conductivity results in Figure 4. The absolute value of the conductivity gap Δ*σ* $\left(\sigma_{cool}^{min}-\sigma_{heat}^{min}\right)$ (Table 3) is largest for samples containing high amounts of filler, simply because at higher loadings the conductivity is higher. The relative comparison of the conductivities, though $\left(\sigma_{cool}^{min}/\sigma_{heat}^{min}\right)$ (Table 3), indicates a maximum hysteresis effect in the range 2.6–4.5 vol% for GNP M−25.

During melting, there is a local density fluctuation on the crystalline regions of the polymer, as these regions are denser than the amorphous part of the matrix. These density changes can dislocate the graphite filler from its position, thus decreasing the overall conductivity of the entire composition. As the temperature increases, increasingly more crystals are melted until the entire matrix is fully amorphous. In this situation, the graphite sheets are less restricted by the polymer matrix and can obtain a more favourable configuration. The fact that graphite can be seen as a two-dimensional semi-metal incorporated in a polar matrix (EVA is polar) indicates that a reduction of the interfacial area can lead to a further reduction of the overall free energy of the system. Having said that, one must not forget that there is a high energy penalty to be paid by the graphite sheets in order to exclude the entire polymer from the interface and achieve full aggregation (thus minimising their surface area). Therefore, one can expect that the energetically favourable configuration is a compromise between these two extremes, manifested in the form of a T shape or various degrees of overlapping platelets, where graphite sheets are arranged in a way that they are “touching” each other, allowing electronic flow. As long as the conductive units do not build cyclic forms, something that can be avoided by sufficiently high loading, they can maintain the integrity of the conductive network in the melt.

**Figure 6:**
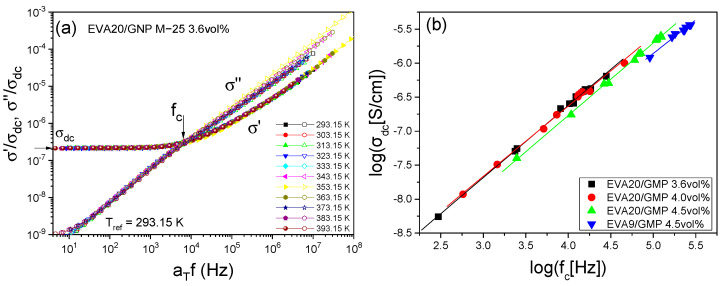
(**a**) AC conductivity master plot for the data of EVA with 3.6 vol% GNP in the temperature range 293.15–393.15 K. The isotherms superpose rather well over the isotherm of reference, by using the same horizontal and vertical shift factors for both *σ*′ and *σ*″. The crossing frequency was used for extracting fc before shifting. (**b**) BNN plot: log*σ_dc_* vs. log(*f_c_*) for the graphene composites as indicated. The solid lines are linear regressions of the data (all linear coefficients 1.00 ± 0.02).

The dielectric spectra are depicted in Figure 6a as a function of reduced frequency for several temperatures in the range from 293.15 to 393.15 K relative to the reference temperature (293.15 K). The figure displays the real and imaginary parts of the conductivity [27]. This representation emphasises the transition from the disperse AC conductivity at higher frequencies to DC conductivity at lower frequencies, according to the linear response theory [60]. Notice that, by employing the time–temperature-superposition principle (tTs), the master curve spans an extended frequency range compared to the actually measured. The charge carrier motion is provided from the crossing of the real and imaginary parts at *f_c_*. As shown in Figure 6b, for representative compositions, the Barton–Nakajima–Namikawa (BNN) relationship [61] dictates that *σ_dc_*~*f_c_*, is obeyed in all cases. Irrespective of the filler loading or polymer type, the slope is close to unity in the double logarithmic plot. This supports the assumption that the conduction mechanism is independent of the ion concentration.

**Figure 7:**
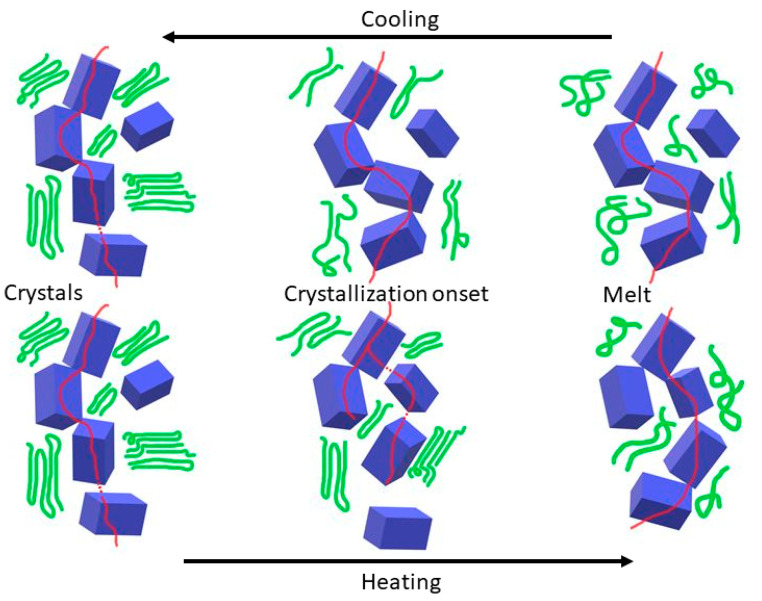
Schematic representation of the integrity of the GNP network (blue) under cooling and heating, indicating the crystalline phase (green) and the percolative network (red line).

Upon cooling, the overall conductivity of the system remains almost constant. On the onset of crystallisation, a large number of nucleating sites can be present, and the eventual crystals grow in a restricted environment created by the graphite platelets. The platelets can be dislodged from their position by the growing crystals, but due to their small size, the GNP sheets can rearrange themselves in order to restore the integrity of the conductive network. As crystallisation continues, the growing crystals are forced by the platelets to adopt a more two-dimensional geometry (see thermal analysis below). Secondary crystallisation is expected to have less impact on the overall conductivity, as it takes place in the amorphous region and the growth of many small crystals disturbs the surrounding conductive network less than during the melting process. The schematic in Figure 7 illustrates these ideas for the benefit of the reader.

### 3.3. Thermal Properties

In Table 4, the thermal properties in terms of the crystallisation and melting temperatures of the CB and GNP-based compounds can be seen. 

Addition of CB particles to EVA20 causes both crystallisation and the melting temperatures to be reduced, up to two and three degrees, respectively. This behaviour clearly excludes a heterogeneous nucleation effect of CB. Primary and secondary nucleation are therefore affected by the presence of CB, likely hindering macromolecular chain mobility and, hence, reducing the frequency of repeating units’ arrival at the growing nucleus or front. The decrease in melting temperature also points towards a somewhat lower thickness of the lamellae formed. Therefore, filler particles can influence crystallisation by creating a somewhat confined environment with fewer density fluctuations, causing a higher degree of supercooling required for nucleation. In the case of EVA20, since both melt and crystallisation temperatures are reduced, the hindering of the chain mobility appears to be the dominant factor.

The crystallisation temperatures instead increase with the addition of small amounts of GNP to EVA20 and remain almost constant with increasing filler loading. An increase in the crystallisation temperature suggests that the crystallisation process is faster with the filler introduction, although there appears to be a limit, as the temperature reaches a plateau fairly quickly. In general, for a nucleating agent, a proportional increase of TC as a function of filler loading is expected, something that is clearly not the case for the GNP-based system. The crystallisation temperature remains largely unchanged, indicating that the number of nucleation sites does not change with increasing GNP amounts into the system. This effect is strongly dependent on the specific interactions between polymer and filler, and for a system of poly(vinylidene fluoride)/GNP where TC increases proportionally with the filler loading, it has been suggested that the graphite platelets act as nucleation origins for a process of heterogeneous nucleation [28]. For the EVA20/GNP system, it appears that the nucleation process is either homogeneous or takes place away from the filler surface. This effect can be further attributed to the lack of appropriate lattice matching between the polymer and the GNP surface. In the ideal case, the polymer can use the flat filler surface as a nucleation site and the only restriction is competition between different crystals growing in overlapping directions. This would lead to a situation where crystals can grow essentially perpendicular to the filler’s surface, as this is the only structural arrangement with the least energetic restrictions. In this work, the large agglomerations of the GNP particles create uneven surfaces that restrict the non-overlapping crystal growth directions arising from the surface-located nucleation sites.

**Figure 8:**
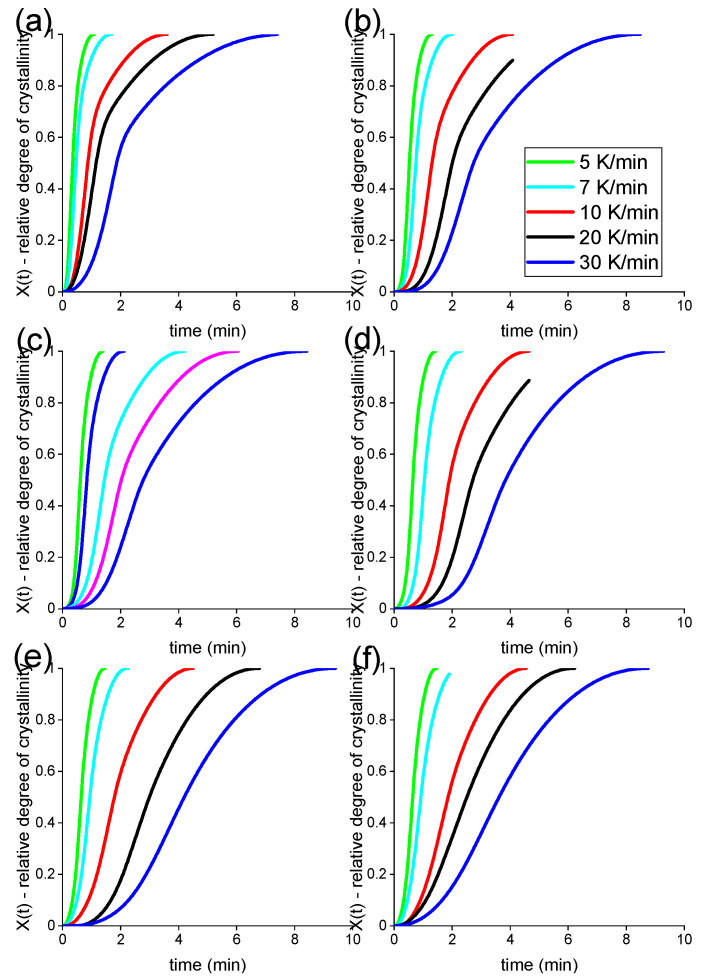
Relative crystallinity as a function of time for pure EVA20 (**a**) and its CB-based composites with loadings of 1.7 vol% (**b**), 4.5 vol% (**c**), 11.5 vol% (**d**), 21.2 vol% (**e**) and 25.8 vol% (**f**) at cooling rates between 5 K/min and 30 K/min.

**Figure 9:**
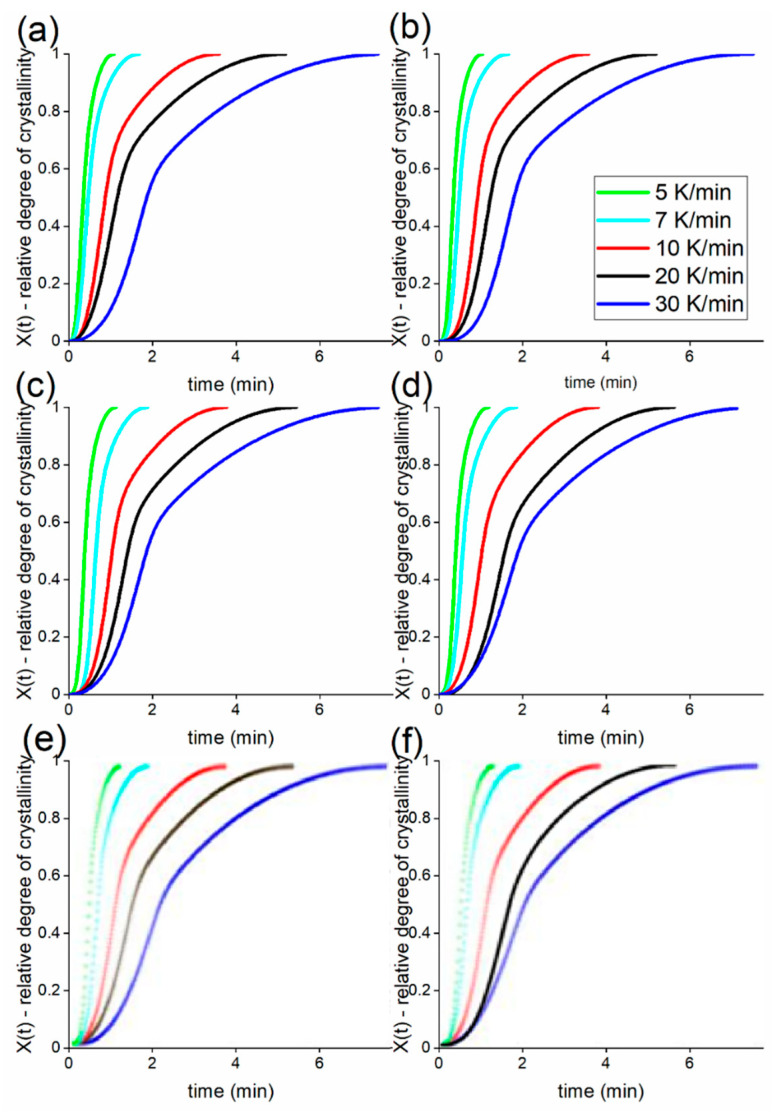
Relative crystallinity as a function of time for pure EVA20 (**a**) and its GNP-based composites with loadings of 0.2 vol% (**b**), 1.7 vol% (**c**), 3.6 vol% (**d**), 4.5 vol% (**e**) and 6.5 vol% (**f**) at cooling rates between 5 K/min and 30 K/min.

In order to check this hypothesis further, additional DSC experiments were performed at different cooling rates. Indeed, semi-crystalline polymers solidify to different extents depending on the cooling rate experienced in processing conditions. The information from thermograms obtained with the DSC at different cooling rates can, however, be extrapolated to higher cooling rates, at least until a different sort of quenching takes place. These methods normally allow one to highlight differences in the solidification behaviour that standard DSC conducted at 10 K/min is not able to reveal completely. In all cases considered, unexpectedly, the crystallisation behaviour was apparently not affected by the presence of fillers, as shown in Figure 8 and Figure 9, as calculated using Equations (3) and (4). These plots show the development of relative crystallinity, or degree of space filling, at five different cooling rates in a pseudo-Avrami fashion.

In Figure 8 and Figure 9, the development of the degree of space filling for five different EVA20/CB and five EVA20/GNP compositions, together with the pure EVA20, can be seen at five different cooling rates ranging between 5 K/min and 30 K/min. Table 5 and Table 6 report the apparent half times of crystallisation for the compounds. A larger amount of CB incorporated in the EVA20 matrix increases the half time of crystallisation at each cooling rate, leading to an apparent reduction of the crystallisation rate of the composite. Therefore, it appears that the reduction of the crystallisation rate is largely due to the hinderance of the polymer diffusion in the crystallisation front caused by the presence of CB aggregates and agglomerates. The half time of crystallisation for the EVA20/GNP systems is instead almost unchanged in comparison to the pure EVA20, confirming the results in Table 4.

#### VA Content

**Figure 10:**
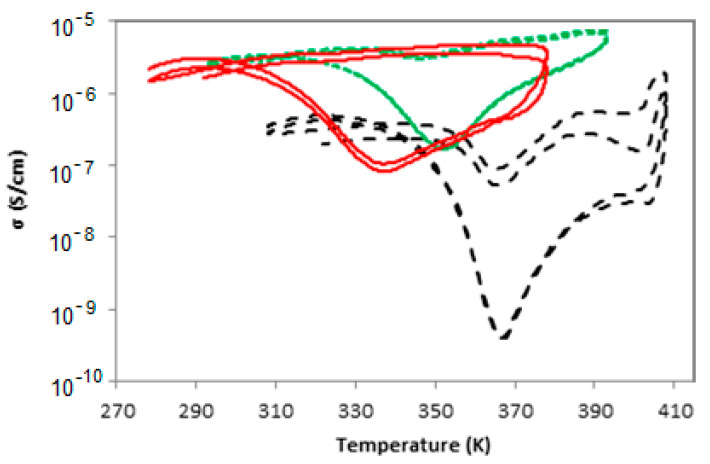
Thermal cycling of EVAs with 4.5 vol% GNP and increasing VA content: 9% (dashed black line), 20% (dotted green line) and 33% (continuous red line). Notice the shift of the conductivity minimum to a lower temperature with increasing VA content.

In Figure 10, the conductivity as a function of temperature for EVA9/GNP, EVA20/GNP and EVA33/GNP can be seen. All systems are filled with 4.5 vol% GNP in order to facilitate comparisons. Since the different polymers have different melting and crystallisation temperatures, the temperature range of each thermal cycling was shifted appropriately to guarantee that the sample is completely molten at the annealing point. For all systems, the temperature range was kept constant, with the annealing point located at 40 K above the melting temperature for the individual component, as measured by DSC.

The systems with higher VA content exhibit higher conductivity, something that can be connected to the lower degree of crystallinity, as seen by DSC. The minimum in the measured conductivity was observed at lower temperatures for the samples containing high VA amounts. Upon cooling, the minimum is observed at a temperature approximately 8 K higher than *T_c_* and, upon heating, the minimum conductivity is observed approximately 3 K lower than the melting temperature. This behaviour suggests a connection between the phase transition and the almost stable value of conductivity upon cooling for these EVA-based systems. The most probable reason why the minimal conductivity is not observed precisely at the phase transition temperatures is most likely linked to the polydispersity of the samples. The systems containing 9% VA and 20% VA have a polydispersity of 13.1 and 11.5, respectively, with the sample containing 33% VA having a polydispersity of 4.4. In the case of the material with low polydispersity, the conductivity minimum is observed at almost the same temperature as the melting or crystallisation temperature (367 K and 365 K), whereas for the systems of larger polydispersity, the differences were somehow larger (336 K and 319 K for 33% VA and 353 K and 348 K for 20% VA). In Table 7, the NTC and PTC values observed for these systems can be seen. The absolute conductivity gap appears to increase with increasing VA content, but both the quotient of the minimal conductivities and the temperature coefficients decrease with increasing VA content. In the case of EVA33, the overall conductivity upon cooling appears to be almost unchanged for both cycles, something seen from the PTC and NTC values (Table 7). Consequently, the effect that causes the differences in conductivity upon cooling and heating appears to be largest for EVA33. Based on the previous discussion for EVA20/GNP systems, it is possible that, for EVA33, the filler–polymer interaction is very weak, thus leaving the conductive network almost unchanged during the crystallisation process. One possible reason for such behaviour may be the significantly lower crystallinity of EVA33, which results in the total volume change caused by the crystal formation being minimal. According to this, the high VA content is probably leading to a reduction of nucleating activity that can cause the crystallisation process to occur far from the graphitic platelets. In the literature, it has been suggested that there is a change in morphology with increasing VA content [62]. From the results of this study, there is no evidence that the change in morphology has a significant impact on the overall conductivity of the system, especially the behaviour upon cooling. While the drop is largest in the EVA9 system, there is a clear trend in the VA content range and an almost infinitesimal change in the conductivity upon cooling, despite the sudden morphological changes occurring in the system as it moves from the melt to the semi-crystalline phase.

The level of crystallinity of the GNP-based system is always smaller than the one observed for the equivalent pure polymer. In the case of EVA20 and EVA33, the crystallinity difference from the equivalent 4.5 vol% GNP composition is almost 2%, whereas in the case of EVA9, the same difference is increased to approximately 4%. This kind of difference can be attributed to the different way the GNP platelets interact with the EVA9, probably due to the small amount of VA groups that allow the polymer to crystallise closer to the graphitic platelets. Furthermore, it has been reported that an EVA with 9% VA has a different morphology from systems with 20 or 33% VA [62].

The existence of CB and GNP fillers in the EVA matrix has an effect on the morphology and electrical behaviour of the system, something already seen in a recent study of a blend of EVA with CB and ethylene-propylene copolymer [59]. In another recent study where graphene nanosheets were introduced to poly(L-lactide) (PLLA), the half time for crystallisation was seen to significantly reduce in comparison with the pure matrix [63]. This behaviour was attributed to a size-dependent soft epitaxy mechanism, where crystallisation kinetics are heavily influenced by the dimensionality of the filler. According to this, for one-dimensional systems such as carbon nanotubes, the polymer has been seen to preferentially align along the tube axis, with no lattice matching between the filler surface and the polymer matrix. For a case of a 2D structure such as GNP, the lattice matching is the dominant force in crystallisation, as the polymer chains need to be absorbed on the platelet surface, therefore undergoing structural changes and requiring more time for the crystallisation process to occur [5]. The graphitic surface available for crystallisation being flat allows the polymer crystals to grow on multiple points and with multiple orientations. It has been speculated that adjacent crystals could interfere during their growth phase and suppress the whole crystallisation kinetics of the system [63]. Similar results have been reported for GNP-based systems of isotactic polypropylene (iPP), where *α* and *β* phases are heavily influenced by the existence of the filler [29]. In this work, the existence of GNP indicates an almost inert system where crystallisation occurs away from the filler, although morphological changes are present. The main reason for this discrepancy can be traced to the preparation method of the composites and the extensive agglomeration and aggregation present in the system. The platelet intercalations lead to an overall filler of a lower aspect ratio than those reported for individual platelets with a more random shape. Since the lattice matching between filler and polymer has been seen to play a vital role in the crystallisation of these types of systems, one can expect that the overall crystallisation is severely hindered by the random orientation of the individual platelets. This leads to individual crystals trying to grow at multiple points, with multiple orientations, from every graphitic surface that has access to the polymer matrix. Obviously, since the platelets are heavily aggregated, these crystallisation efforts are very expensive energetically and, thus, highly improbable. Furthermore, it has been predicted that the overall morphology and absorption with the subsequent lamellar orientation in the case of a graphene-based composite is a highly cooperative process [64,65], and in the case of systems prepared with solution mixing, GNP has been reported to facilitate crystallisation [5,26,28].

## 4. Conclusions

This study showed a detailed analysis of the electrical and thermal behaviour of a nanocomposite system comprised of EVA20 and CB or GNP. Temperature-dependent broadband dielectric spectroscopy was used in an effort to evaluate the overall conductivity of the system as a function of the amount of conductive filler, as well as the polymer and relaxation dynamics and ion mobility. Further analysis using DSC was performed yielding detailed information on the mechanisms behind the crystallisation process and the polymer–filler interactions during primary, secondary and (where present) tertiary crystallisation.

The CB-based systems exhibited an initial electrical percolation threshold at 2.2 vol%. Electrical hysteresis as a function of temperature and filler loading was measured with the use of positive and negative temperature coefficients. For systems with filler amounts close to the percolation threshold, the maximum value in the temperature coefficients was observed. Both melting and crystallisation temperatures are affected by the presence and the amount of CB, decreasing in a proportional manner with the filler load. Despite this, the change in temperature was in the order of a few degrees, indicating that CB particles weakly affect the crystallisation process. Further analysis indicated that CB particles are slightly active as nucleating agents in small quantities, but the effect reaches a plateau fairly quickly, with increasing filler becoming more inert. It is believed that aggregation and agglomeration of the particles increase the effective size, thus dispersing the overall effect. The crystallisation process was found to be a three-stage process, with initial fibrillar growth followed by a spherulitic overall arrangement. In comparison, it is important to stress that pure EVA is dominated by lamellar to spherulitic growth at low crystallisation rates.

GNP-based systems exhibited a far lower percolation threshold of the order of ~5 vol%. On the contrary to CB-based systems, GNP nanocomposites exhibited an almost negligible amount of electrical hysteresis upon cooling, both above and below the percolation threshold. This behaviour was attributed to the capability of the filler to maintain the integrity of the conductive network by restricting the onset of crystallisation to regions where no filler is present. Therefore, the polymer dynamics remain largely unaffected and follow the Dyre model, which assumes charge transport occurs by hopping of charge carriers in the spatially varying random energy landscape. The almost instantaneous increase and reaching of a crystallisation plateau and melt temperature, even at very small amounts of GNP, indicated that the fillers do not act as nucleating agents, but are located in the amorphous part of the matrix. Primary crystallisation was found to be dominated by lamellar-spherulitic growth, followed by a secondary crystallisation in a manner similar to the pure EVA.

## Figures and Tables

**Table 1:** Main characteristics of the EVAs used in this study.

Polymer	Sample Code	Vinyl Acetate Content (%)	Melting Temperature (°C)	Density (kg/m^3^)	M_w_ (kg/mol)	M_w_/M_n_
Escorene™ Ultra FL 00909	EVA9	9	94	928	184	13.1
Escorene™ Ultra UL 02020	EVA20	20	80	940	161	11.5
Escorene™ Ultra UL 04533EH2	EVA33	33	61	956	n.a.	4.4

**Table 2:** Characteristics of the conductivity as a function of temperature for the EVA20/CB systems.

CB (vol%)	NTC_cool_	PTC_cool_	NTC_heat_	PTC_heat_	$\sigma_{cool}^{min}-\sigma_{heat}^{min}$	$\sigma_{cool}^{min}/\sigma_{heat}^{min}$
11.4	2	35	297	5398	1.177 × 10^−5^	138
16.0	1	19	12	174	4.128 × 10^−5^	8
21.0	1	57	6	276	1.018 × 10^−4^	4
22.5	1	47	4	173	1.304 × 10^−4^	3
25.5	1	84	2	119	3.650 × 10^−5^	1
29.7	2	110	1	13	−2.955 × 10^−4^	1

**Table 3:** Characteristics of the conductivity as a function of temperature for the EVA20/GNP systems.

GNP (vol%)	NTC_cool_	PTC_cool_	NTC_heat_	PTC_heat_	$\sigma_{cool}^{min}-\sigma_{heat}^{min}$	$\sigma_{cool}^{min}/\sigma_{heat}^{min}$
1.7	1	1	2	3	3.501 × 10^−12^	2
2.6	1	1	17	10	2.309 × 10^−10^	12
3.5	3	0	1601	201	2.537 × 10^−7^	558
4.5	2	1	489	204	2.473 × 10^−6^	241
5.5	1	1	17	20	8.176 × 10^−6^	15
6.4	2	1	9	6	2.419 × 10^−5^	6

**Table 4:** Melting (*T_m_*) and crystallisation (*T_c_*) temperatures for the various compositions of EVA20 as a function of filler loading as observed by DSC.

GNP (vol%)	0	1.7	4.5	11.4	21	25.6			
*T_c_* (K)	340.2	340.8	340.7	339.9	338.7	338.6			
*T_m_* (K)	356.7	356.0	355.5	355.0	354.1	353.8			
GNP (vol%)	0.2	0.4	0.9	1.7	2.6	3.5	4.5	5.5	6.4
*T_c_* (K)	339.5	339.6	339.8	340.1	340.4	340.2	340.5	340.6	340.5
*T_m_* (K)	357.2	357.0	357.4	357.2	357.1	356.8	357.0	357.1	357.1

**Table 5:** Apparent half times t_1/2_ of the EVA20/CB composites’ crystallisation process at different cooling rates. The pure EVA20 noted as 0 vol% is displayed for comparison.

CB (vol%)	5 K/min (min)	7 K/min (min)	10 K/min (min)	20 K/min (min)	30 K/min (min)
0	1.6	1.2	0.9	0.5	0.3
1.7	2.7	2.0	1.3	0.8	0.5
4.5	2.8	2.0	1.4	0.8	0.6
11.5	3.8	2.7	1.9	1.1	0.6
21.2	4.2	3.0	1.8	0.9	0.6
25.8	3.6	2.5	1.9	0.9	0.6

**Table 6:** Half times t_1/2_ of the EVA20/GNP composites’ crystallisation process at different temperature gradients. The pure EVA20 noted as 0 vol% is displayed for comparison.

GNP (vol%)	5 K/min (min)	7 K/min (min)	10 K/min (min)	20 K/min (min)	30 K/min (min)
0	1.6	1.2	0.9	0.5	0.3
0.2	1.8	1.2	0.9	0.5	0.3
1.7	1.9	1.4	1.0	0.9	0.4
3.6	1.9	1.6	1.0	0.6	0.4
4.5	2.2	1.5	1.1	0.6	0.4
6.5	2.0	1.8	1.1	0.6	0.5

**Table 7:** Temperature coefficients for the different EVAs at 4.5 vol% GNP loading.

%VA	NTC_cool_	PTC_cool_	NTC_heat_	PTC_heat_	$\sigma_{cool}^{min}-\sigma_{heat}^{min}\;(S/\mathrm{cm})$	$\sigma_{cool}^{min}/\sigma_{heat}^{min}$
9	3	16	843	4434	1.09 × 10^−7^	271
20	1	2	179	420	2.44 × 10^−6^	221
33	0	1	21	35	5.02 × 10^−6^	49

## Supplementary Materials

The following supporting information can be downloaded at: https://www.mdpi.com/article/10.3390/nano12203602/s1, Figure S1: DSC traces of EVA20 and an EVA/GNP composite.; Figure S2: Comparison of dielectric loss spectra of the pure EVA20 and an EVA/GNP composite.
