# Supplementary material for: Crystallisation Kinetics and Associated Electrical Conductivity Dynamics of Poly(Ethylene Vinyl Acetate) Nanocomposites in the Melt State
Source: Nanomaterials (Basel). 2022 Oct 14;12(20):3602. doi: 10.3390/nano12203602 (PMC9612297; doi:10.3390/nano12203602)
Supplement: Supplementary file 1 [file nanomaterials-12-03602-s001.zip › nanomaterials-1908776-supplementary.pdf]

nanomaterials

Supporting Information

Crystallisation Kinetics and Associated Electrical Conductivity Dynamics of Poly(Ethylene Vinyl Acetate) Nanocomposites in the Melt State

Gertrud Stalmann [1,2,3], Aleksandar Matic [1], Per Jacobsson [1], Davide Tranchida [4], Antonis Gitsas [4,*] and Thomas Gkourmpis [5,*]

[1] Department of Applied Physics, Chalmers University of Technology, 412 96 Gothenburg, Sweden
[2] Department of Physics, University of Gothenburg, 405 30 Göteborg, Sweden
[3] Department of Physics, Philipps-Universität Marburg, 35037 Marburg, Germany
[4] Innovation & Technology, Borealis Polyolefine GmbH, 4021 Linz, Austria
[5] Innovation & Technology, Borealis AB, 444 86 Stenungsund, Sweden
* Correspondence: antonis.gitsas@borealisgroup.com (A.G.); thomas.gkourmpis@borealisgroup.com (T.G.); Tel.: +43-732-6981-5739 (A.G.); +46-303-205-576 (T.G.)

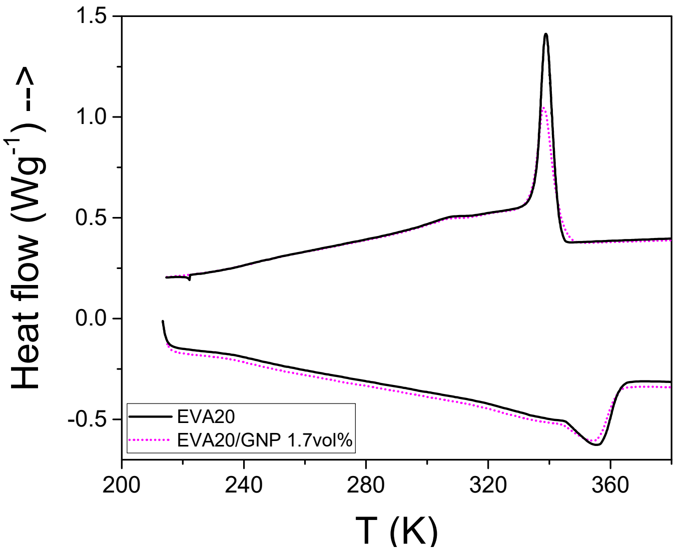

Figure S1. DSC traces of EVA20 (solid line) and an EVA/GNP composite (dotted line) obtained during the second heating and cooling runs (rate 10 K/min), exhibiting, respectively, an exothermic peak (crystallization with heats of fusion of 104 & 97 J/g) and an endothermic peak (melting with heats of fusion of 105 and 100 J/g, respectively).

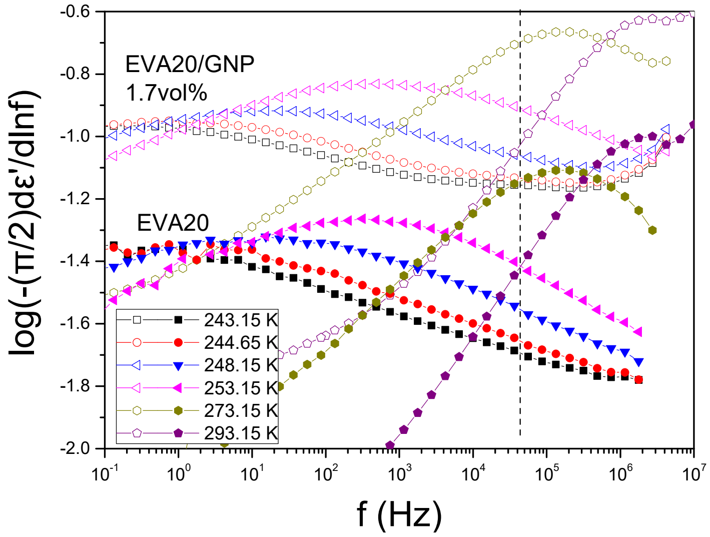

Figure S2. Comparison of dielectric loss spectra of the pure EVA20 (filled symbols) and an EVA/GNP composite (open symbols) at representative temperatures. The composite being close to the conductivity threshold leads to an increase of the dielectric intensity but not to a speed-up of the dynamics. Note that the position of the secondary, local relaxation shoulder observed at higher frequencies marked with the dashed line is also not significantly affected in the composite.
